# Timeliness of Broad-Spectrum Antibiotic De-escalation and Mortality in Emergency Department Patients With Sepsis: A Retrospective Hierarchical Logistic Regression Study

**DOI:** 10.7759/cureus.90728

**Published:** 2025-08-22

**Authors:** Amin Farsani, Pardeep Thandi, Zin Htway

**Affiliations:** 1 Emergency Medicine, Los Robles Regional Medical Center, Thousand Oaks, USA; 2 Pathology, Los Robles Regional Medical Center, Thousand Oaks, USA

**Keywords:** antibiotic de-escalation, broad-spectrum antibiotics, mortality after sepsis, narrow-spectrum antibiotics, severe sepsis

## Abstract

Background

Early initiation of broad-spectrum antibiotics (BSA) is a cornerstone of sepsis management; however, prolonged use without timely de-escalation to narrow-spectrum antibiotics (NSA) can contribute to adverse outcomes, including higher mortality and antimicrobial resistance. While intensive care unit (ICU) studies support the benefits of antibiotic de-escalation, its timing and impact in emergency department (ED) settings remain underexplored.

Objective

To examine the association between delayed de-escalation from BSA to NSA and in-hospital mortality among ED patients with sepsis, adjusting for the Charlson Comorbidity Index (CCI), age, and sex.

Methods

We conducted a retrospective multicenter cohort study of adult ED patients (≥18 years) hospitalized with sepsis between January 2021 and December 2023. All received empiric BSA followed by de-escalation to NSA. De-escalation timing was measured in 6-hour intervals starting 61 minutes after BSA initiation. Hierarchical binary logistic regression assessed associations with in-hospital mortality, controlling for CCI, age, and sex.

Results

Among 2,906 eligible patients, delayed de-escalation was independently associated with increased mortality (OR per 6-hour delay = 1.006; 95% CI, 1.001-1.012; *p* = .023). Higher CCI and age were also significant predictors (CCI OR = 1.175; age OR = 1.014; both *p* ≤ .001). Sex was not significantly associated with mortality (*p* = .502). Each six-hour delay beyond the first hour increased the odds of death by approximately 0.6%.

Conclusion

Delayed de-escalation of empiric BSA in ED sepsis patients is linked to higher in-hospital mortality, reinforcing the need for prompt reassessment and narrowing of antibiotic therapy as part of ED antimicrobial stewardship.

## Introduction

Sepsis is a life-threatening condition characterized by a dysregulated host response to infection, leading to organ dysfunction and high mortality rates globally, particularly in emergency and critical care settings [1]. The burden of sepsis has increased significantly over the past two decades, with recent US data showing rising incidence and mortality [2-5]. This trend is further complicated by the aging population and increasing prevalence of chronic comorbidities [6-8].

Prompt administration of empiric broad-spectrum antibiotics is a cornerstone of sepsis management and is associated with improved survival [9]. In the US, the Centers for Medicare & Medicaid Services (CMS) implemented the SEP-1 (Severe Sepsis and Septic Shock Early Management Bundle) core measure, which mandates early identification of sepsis and initiation of treatment bundles (including broad-spectrum antibiotics) within three hours of ED presentation [10]. While SEP-1 emphasizes early initiation, it does not address the timing of subsequent de-escalation, leaving a critical stewardship gap. Prolonged exposure to broad-spectrum antibiotics can lead to antimicrobial resistance, adverse events, *Clostridioides difficile* (*C. difficile*) infection, and unnecessary healthcare costs [11-13]. Therefore, antimicrobial stewardship programs emphasize timely de-escalation from broad-to-narrow spectrum agents (B2N) based on clinical and microbiological data [14,15].

While de-escalation has been associated with improved outcomes in ICU settings [16-18], data are limited regarding its timing and direct impact on mortality in emergency department (ED) populations. Unlike ICU settings, where patients often have prolonged hospitalizations and continuous monitoring, the ED environment is marked by rapid turnover, diagnostic uncertainty, and variability in clinician decision-making. As such, findings from ICU or infection-specific studies may not generalize well to ED settings, where time-sensitive decisions regarding antimicrobial management are made in the absence of definitive culture data. As the frontline of hospital care, the ED represents a critical yet understudied setting for stewardship interventions targeting sepsis management. Previous research has largely examined specific antimicrobial administration strategies, appropriateness of therapy, or outcomes in narrowly defined infections such as healthcare-associated pneumonia [19-21], rather than addressing the broader impact of de-escalation timing on overall mortality in undifferentiated sepsis presentations typical of the ED.

This study addresses that gap by evaluating the association between time to de-escalation and in-hospital mortality in ED patients with sepsis. We hypothesize that delays in de-escalation, even after early empiric therapy, are independently associated with worse outcomes. To account for potential confounding, we adjusted for age, sex, and comorbidity burden using the Charlson Comorbidity Index (CCI), a validated tool for prognostic risk adjustment in clinical research [22]. A total of 2,906 patients met the inclusion criteria for this analysis. Our findings aim to inform real-time antimicrobial decisions in the ED and reinforce the importance of stewardship protocols [23-26].

Emerging research has emphasized not only the importance of early antibiotic initiation but also the potential mortality benefit of early de-escalation. Seymour et al. demonstrated that delays in initial antibiotic administration increased mortality in patients with septic shock [27]. Similarly, Kam et al. found that de-escalation by hospital day four in patients with suspected sepsis was associated with reduced risk of ICU admission, acute kidney injury, and mortality [28]. Despite these findings, a Cochrane review identified a persistent lack of randomized trials examining the safety and efficacy of de-escalation timing in sepsis, underscoring the value of ED-focused observational research like the present study [29].

## Materials and methods

Study design and setting

This retrospective multicenter cohort study was conducted from January 2021 to December 2023 at multiple HCA (Hospital Corporation of America) hospitals across the country. It included 2,906 adult patients (≥18 years) who presented to the emergency department (ED) with suspected or confirmed infection, received empiric broad-spectrum antibiotics (BSA), and underwent documented de-escalation to narrow-spectrum antibiotics (NSA) during hospitalization. Sepsis was defined according to the CMS SEP-1 measure [10]. Patients met criteria if they had suspected or confirmed infection and at least two systemic inflammatory response syndrome (SIRS) criteria [10]. De-escalation to narrow-spectrum antibiotics (NSA) was defined as a documented change from empiric broad-spectrum therapy to a more targeted regimen. Indications for de-escalation in participating hospitals followed routine clinical practice and were not protocolized for this study. Treating teams typically narrowed therapy based on one or more of the following: (1) microbiological results demonstrating a susceptible pathogen, (2) clinical improvement with stabilization of vital signs or laboratory parameters, (3) identification of a specific infection source allowing targeted therapy, or (4) recommendations from antimicrobial stewardship or infectious diseases consultation. Our analysis reflects the timing of de-escalation as it occurred in real-world practice rather than by predetermined study criteria. Inclusion required accurate documentation of both antibiotic initiation and de-escalation times. Patients were excluded if they died within 60 minutes of BSA administration, had missing or incomplete antibiotic timing data, or were transferred from outside facilities without verifiable antibiotic records.

Variables

The present study predicted mortality (yes/no) from time to de-escalation, measured in six-hour intervals beginning at 61 minutes after initial BSA administration while controlling for the CCI (continuous), age (continuous), and sex (binary).

Statistical analysis

Mortality was predicted utilizing a hierarchical binary logistic regression in four blocks using the IBM SPSS Statistics for Windows, version 29 (IBM Corp., Armonk, NY, USA). Model 1 assessed the association between de-escalation timing and mortality. Models 2 through 4 sequentially adjusted for CCI, age, and sex. Odds ratios (OR), 95% confidence intervals (CI), and two-sided p-values were reported. A p-value of <0.05 was considered statistically significant. McFadden’s pseudo R^2^ (MFPRS) and log likelihood (-2LL) were used as tools for evaluating how well a logistic regression model explains variance in the outcome.

## Results

To investigate the association between B2N time, which is the time it took to de-escalate from broad-spectrum to narrow-spectrum antibiotics, and mortality when considering CCI score, patient age, and patient gender, a hierarchical binary logistic regression analysis was conducted using IBM SPSS Statistics for Windows, version 29 (IBM Corp., Armonk, NY, USA).

**Figure 1:**
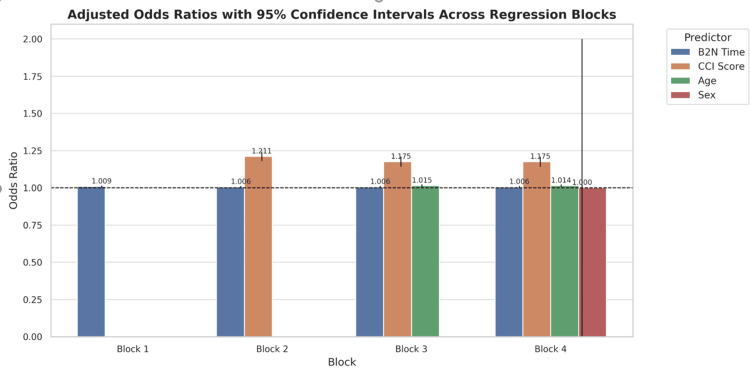
Bar chart with 95% confidence intervals and odds ratio (OR) annotations for each predictor across blocks. This figure visually conveys the precision of estimates and the relative contribution of each variable. The dashed line at OR = 1 represents the threshold for no effect. CCI: Charlson Comorbidity Index; B2N: broad-to-narrow antibiotic de-escalation.

For Block 1, B2N time and mortality were analyzed. B2N time was found to be a significant positive predictor of mortality (B = .009, SE = .003, Wald = 10.723, df = 1, Exp(B) = 1.009, 95% CI (1.003, 1.014), p = .001) (Table 1, Figure 1).

**Table 1:** Hierarchical logistic regression results. The results of hierarchical binary logistic regression models analyzing the association between time to broad-to-narrow antibiotic de-escalation (B2N) and in-hospital mortality among emergency department patients with sepsis. Covariates were added across four model blocks to assess the adjusted impact of B2N time while controlling for Charlson Comorbidity Index (CCI), patient age, and sex.

Model block	Variable	B	SE	Wald	df	p-value	Odds ratio	95% CI lower	95% CI upper
Block 1	B2N time	0.009	0.003	10.723	1	0.001	1.009	1.003	1.014
Block 2	B2N time	0.006	0.003	5.400	1	0.020	1.006	1.001	1.012
	CCI	0.191	0.013	206.166	1	<0.001	1.211	1.18	1.243
Block 3	B2N time	0.006	0.003	5.135	1	0.023	1.006	1.001	1.012
	CCI	0.161	0.015	115.298	1	<0.001	1.175	1.141	1.21
	Age	0.014	0.003	19.452	1	<0.001	1.015	1.008	1.021
Block 4	B2N time	0.006	0.003	5.133	1	0.023	1.006	1.001	1.012
	CCI	0.161	0.015	115.476	1	<0.001	1.175	1.141	1.21
	Age	0.014	0.003	19.254	1	<0.001	1.014	1.008	1.021
	Sex	0.057	0.086	0.451	1	0.502	1.059	0.896	1.253

**Figure 2:**
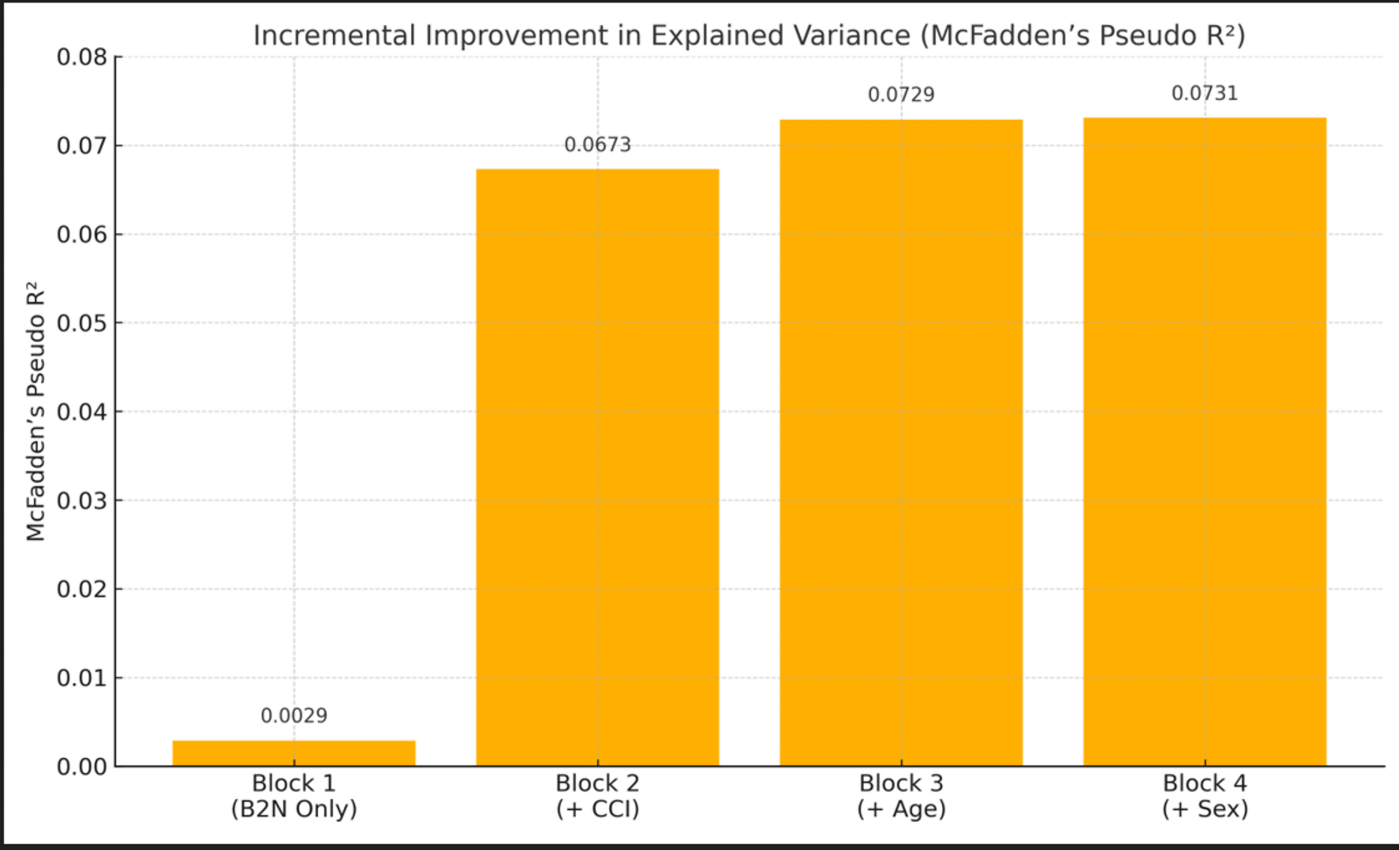
Incremental improvement in variance explained (McFadden’s pseudo R²) across hierarchical regression blocks. The greatest increase in explanatory power occurs with the inclusion of Charlson Comorbidity Index (CCI) in Block 2. Subsequent additions of age and sex yield modest and negligible gains, respectively. The final model explains 7.3% of mortality variance, modest but acceptable for clinical prediction in complex systems like sepsis care. B2N: broad-to-narrow antibiotic de-escalation.

The analysis suggests that for every six-hour delay in B2N time following the first 60 minutes, the odds ratio for mortality increases by approximately one percent. The variance explained for this block was McFadden’s pseudo R² (MFPRS) = 0.0029 (Table 2, Figure 2).

**Table 2:** Regression block summary. B2N: broad-to-narrow antibiotic de-escalation.

Block	Variables entered	-2 log likelihood (-2LL)	Δ -2LL from previous block	McFadden’s pseudo R²
Null	None (intercept only)	3545.586	–	–
Block 1	B2N > 60 min within 240 min	3535.157	10.429	0.0029
Block 2	+ Charlson Comorbidity Index (CCI)	3306.855	228.302	0.0673
Block 3	+ Age	3286.992	19.863	0.0729
Block 4	+ Sex	3286.541	0.451	0.0731

For Block 2, the relationship between B2N and mortality was assessed while controlling for CCI score. In this model, B2N time was found to be a significant positive predictor of mortality (B = .006, SE = .003, Wald = 5.400, df =1, Exp(B) = 1.006, 95% CI (1.001, 1.012), p = .020) (Table 1, Figure 1). Controlling for B2N time, CCI score was also found to be a significant positive predictor of mortality (B = .191, SE = .013, Wald = 206.166, df = 1, Exp(B) = 1.211, 95% CI (1.180, 1.243), p <0.001) (Table 1, Figure 1). The analysis suggests that controlling CCI score, for every six-hour delay in B2N time following the first 60 minutes, the odds ratio for mortality increases by approximately one-half percent. Furthermore, the analysis suggests that controlling B2N time, for every one-unit increase in CCI score, the odds ratio for mortality increases by more than 20%. Adding in CCI explained significantly more variance (MFPRS = 0.0673) and improved upon Block 1 (Table 2, Figure 2).

For Block 3, the relationship between B2N and mortality was assessed while controlling for CCI score and age. While controlling for CCI score and age, B2N time was found to be a significant positive predictor of mortality (B = .006, SE = .003, Wald = 5.135, df = 1, Exp(B) = 1.006, 95% CI (1.001, 1.012), p = .023) (Table 1, Figure 1). While controlling for B2N time and age, CCI score was also found to be a significant positive predictor of mortality (B = .161, SE = .015, Wald = 115.298, df = 1, Exp(B) = 1.175, 95% CI (1.141, 1.210), p < 0.001) (Table 1, Figure 1). While controlling for B2N and CCI score, age was also found to be a significant positive predictor of mortality (B = .014, SE = .003, Wald = 19.452, df = 1, Exp(B) = 1.015, 95% C.I. (1.008, 1.021), p < 0.001) (Table 1, Figure 1). The analysis suggests that controlling for CCI score and age, for every six-hour delay in B2N time following the first 60 minutes, the odds ratio for mortality increases by approximately one-half percent. Furthermore, the analysis suggests that controlling for B2N time and age, for every one-unit increase in CCI score, the odds ratio for mortality increases by approximately 17.5%. Lastly, the analysis suggests that controlling for B2N time and CCI score, for every one-year increase in age, the odds ratio for mortality increases by approximately 1.5%. The inclusion of age explained a small but notable gain in variance (MFPRS = 0.0729) from Block 2 (Table 2, Figure 2).

For Block 4, the relationship between B2N time and mortality was assessed while controlling for CCI score, age, and sex. While controlling for CCI score, age, and sex, B2N time was found to be a significant positive predictor of mortality (B = .006, SE = .003, Wald = 5.133, df = 1, Exp(B) = 1.006, 95% CI (1.001, 1.012), p = .023) (Table 1, Figure 1). While controlling for B2N time, age and sex, CCI score was also found to be a significant positive predictor of mortality (B = .161, SE = .015, Wald = 115.476, df = 1, Exp(B) = 1.175, 95% CI (1.141, 1.210), p < 0.001) (Table 1, Figure 1). While controlling for B2N, CCI score, and sex, age was also found to be a significant positive predictor of mortality (B = .014, SE = .003, Wald = 19.254, df = 1, Exp(B) = 1.014, 95% CI (1.008, 1.021), p < .001) (Table 1, Figure 1). While controlling for B2N, CCI score, and age, sex was not found to be a statistically significant predictor of mortality (p = .502). The analysis suggests that controlling for CCI score, age, and sex, for every six-hour delay in B2N time following the first 60 minutes, the odds ratio for mortality increases by approximately one-half percent. Furthermore, the analysis suggests that controlling for B2N time, age, and sex, for every one-unit increase in CCI score, the odds ratio for mortality increases by approximately 17.5%. Lastly, the analysis suggests that controlling for B2N time, CCI score, and sex, for every one-year increase in age, the odds ratio for mortality increases by approximately 1.5%. The addition of patients’ sex was not found to associate with mortality in this last model and provided negligible additional variance (MFPRS = 0.121) from Block 3 (Table 2, Figure 2).

## Discussion

We found that delayed de-escalation of empiric broad-spectrum antibiotics was consistently linked with higher in-hospital mortality, even after adjusting for comorbidities, age, and sex. Each six-hour delay beyond the first hour increased the odds of death by about 0.6%. While statistical modeling showed that comorbidity burden and age explained most of the variation in mortality, the timing of antibiotic de-escalation still had an independent effect. This means that even small delays in narrowing therapy may carry meaningful consequences for patient survival. Overall, the final model explains approximately 7.3% of the variance in mortality (Table 2). Although this may appear modest, it is consistent with expectations for clinical logistic regression models, particularly in complex, multifactorial conditions such as sepsis. Notably, even though B2N timing contributed minimally to overall variance, its independent association with mortality may still be clinically significant in high-acuity emergency care settings. While numerically modest, this effect is clinically meaningful in the high-volume, fast-paced environment of the ED, where even small reductions in mortality can translate into a substantial number of lives saved across large populations. The incremental risk emphasizes the need for time-sensitive clinical decisions and supports integration of time-based de-escalation protocols into emergency workflows, particularly in environments where diagnostic uncertainty may deprioritize stewardship goals. Timely transitions to narrow-spectrum antibiotics may reduce harm associated with BSA overuse, including antimicrobial resistance, adverse events, and *C. difficile* infection [11,30].

Our findings are further supported by recent observational studies. Kam et al. identified that early de-escalation (by hospital day four) reduced the risk of adverse outcomes in patients with suspected sepsis [28]. Zhao et al. additionally found that ICU patients who underwent de-escalation had significantly lower 14-day mortality than those who continued broad-spectrum antibiotics [31]. While our study focuses on the emergency department setting, the consistency of these findings across different settings suggests a broader applicability of timely de-escalation as a stewardship intervention.

Updated guidelines, including those from the Surviving Sepsis Campaign, further support this direction by emphasizing clinical responsiveness and timely stewardship interventions [32,33]. Embedding de-escalation considerations into early sepsis care aligns with these evolving recommendations, which increasingly prioritize reassessment and targeted therapy within hours of presentation.

The urgency of implementing stewardship measures is also informed by the evolving antibiotic pipeline and rising resistance threats. Theuretzbacher discusses scenarios for future antibiotic development, emphasizing the need to preserve existing agents through judicious use [25]. Although the study is more forward-looking, it reinforces the necessity of stewardship initiatives like timely de-escalation [25].

These findings are particularly significant in the context of blood culture processing, which typically takes 24 to 72 hours to yield results [8]. Given this delay, reliance solely on culture data to guide de-escalation may be suboptimal. Clinical judgment, supported by imaging, laboratory trends, and patient response, should guide early narrowing of therapy in appropriate patients [14,34-35]. Blood cultures obtained in the ED are subject to contamination rates between 2% and 3%, which can lead to misinterpretation and unnecessary prolongation of broad-spectrum therapy [36]. Improved procedural training and standardized collection techniques may help mitigate this risk.

Our inclusion of both age and CCI revealed that while CCI includes age as a weighted component [22], age remains an independent predictor of mortality, consistent with prior studies highlighting increased sepsis-related mortality in older adults [7]. Esper and Martin further underscored this relationship by demonstrating how the burden of organ dysfunction significantly contributes to poor outcomes in elderly patients with sepsis, suggesting that age-related physiologic decline may exacerbate vulnerability in this population [8].

Additionally, national surveillance data have documented an alarming rise in sepsis incidence and hospital resource utilization [37-39]. Early de-escalation may offer not only clinical benefit but also system-level efficiencies through reduced length of stay and drug-related complications. Emerging evidence also suggests surrogate parameters, such as *Candida* species burden or host microbiome composition, may aid in early risk prediction and guide antibiotic reassessment [13,23].

Although randomized trials on de-escalation are sparse, noninferiority studies and observational data support its safety and efficacy in ICU and pneumonia-specific cohorts. For example, Leone et al. demonstrated noninferiority in mortality when de-escalation was employed in severe sepsis, while Joung et al. showed no adverse impact on outcomes for ICU-acquired pneumonia [17,18]. Similarly, Ferrer et al. observed clinical benefit in patients with nosocomial sepsis receiving adjusted antibiotic therapy [40]. However, these findings are drawn from highly controlled or infection-specific populations, and their applicability to the ED, characterized by diagnostic ambiguity, limited follow-up, and rapid turnover, remains uncertain. Broader implementation remains challenged by provider reluctance to modify empiric regimens without culture confirmation, even though observational studies and pharmacodynamic analyses support the safety and benefits of early narrowing based on clinical judgment [30,41-43].

Our study’s use of six-hour intervals enabled quantification of a dose-response relationship between delay and mortality, yet further work is needed to define clinically meaningful time thresholds, such as whether de-escalation within 24 or 48 hours offers an optimal balance between safety and stewardship. Subgroup analyses by age and comorbidity burden may reveal differential impacts of delay, particularly in older adults or those with high CCI scores.

De-escalation timing may also be influenced by diagnostic certainty. Infections without culture-positive results or with ambiguous radiologic findings may delay narrowing. Provider behavior, including fear of patient deterioration, defensive medicine, and habitual continuation of initial therapy, can also contribute to delay. Integration of behavioral nudges, such as checklists or real-time decision support, may reduce these barriers.

System-level factors are equally important. Delays may result from operational constraints such as pharmacy turnaround, weekend staffing, or lack of infectious disease consultation. Future studies could examine the effect of time-of-day or day-of-week variations in de-escalation timing and outcomes. Clinical decision support tools embedded in electronic health records (EHRs), pharmacist-led stewardship rounds, or AI-driven reassessment alerts may help standardize and expedite de-escalation in emergency settings.

Finally, prospective multicenter interventional trials are needed to test whether time-targeted de-escalation protocols can reduce mortality and other adverse outcomes. These should examine broader stewardship metrics such as length of stay, antibiotic duration, resistance trends, and cost-effectiveness. Until such trials are conducted, our findings support embedding stewardship principles into emergency care and suggest that earlier reassessment of antibiotic regimens may be lifesaving.

Although our study included 2,906 patients, larger and more diverse cohorts would improve generalizability. We were unable to include race and ethnicity due to incomplete data, and future research should examine whether de-escalation timing impacts outcomes differently across racial and sociodemographic groups. Our study was conducted across multiple centers, which strengthens the generalizability of findings but may also limit region-specific insights. Because healthcare delivery, stewardship practices, and patient demographics vary by location, concentrated analyses within defined geographical areas may yield additional context-specific observations. Future research should examine regional differences to determine whether the relationship between de-escalation timing and outcomes is consistent across diverse healthcare environments.

## Conclusions

Timely de-escalation of empiric antibiotics in the ED is significantly associated with reduced mortality. These findings advocate for embedding antimicrobial stewardship into emergency care practices and highlight the need for prospective studies to establish causal links and optimize de-escalation timing.
